# An Anti-HIV-1 V3 Loop Antibody Fully Protects Cross-Clade and Elicits T-Cell Immunity in Macaques Mucosally Challenged with an R5 Clade C SHIV

**DOI:** 10.1371/journal.pone.0018207

**Published:** 2011-03-31

**Authors:** Jennifer D. Watkins, Nagadenahalli B. Siddappa, Samir K. Lakhashe, Michael Humbert, Anton Sholukh, Girish Hemashettar, Yin Ling Wong, John K. Yoon, Wendy Wang, Francis J. Novembre, Francois Villinger, Chris Ibegbu, Kalpana Patel, Davide Corti, Gloria Agatic, Fabrizia Vanzetta, Siro Bianchi, Jonathan L. Heeney, Federica Sallusto, Antonio Lanzavecchia, Ruth M. Ruprecht

**Affiliations:** 1 Dana-Farber Cancer Institute, Boston, Massachusetts, United States of America; 2 Harvard Medical School, Boston, Massachusetts, United States of America; 3 Yerkes National Primate Research Center, Emory University, Atlanta, Georgia, United States of America; 4 Department of Microbiology and Immunology, Emory University, Atlanta, Georgia, United States of America; 5 Department of Pathology and Laboratory Medicine, Emory University, Atlanta, Georgia, United States of America; 6 Humabs SAGL, Bellinzona, Switzerland; 7 Department of Veterinary Medicine, University of Cambridge, Cambridge, United Kingdom; 8 Institute for Research in Biomedicine, Bellinzona, Switzerland; Karolinska Institutet, Sweden

## Abstract

Neutralizing antibodies have been shown to protect macaques against SHIV challenge. However, genetically diverse HIV-1 clades have evolved, and a key question left unanswered is whether neutralizing antibodies can confer cross-clade protection in vivo. The novel human monoclonal antibody HGN194 was isolated from an individual infected with an HIV-1 clade AG recombinant circulating recombinant form (CRF). HGN194 targets an epitope in the third hypervariable loop (V3) of HIV-1 gp120 and neutralizes a range of relatively neutralization-sensitive and resistant viruses. We evaluated the potential of HGN194 to protect infant rhesus monkeys against a SHIV encoding a primary CCR5-tropic HIV-1 clade C envelope. After high-dose mucosal challenge, all untreated controls became highly viremic while all HGN194-treated animals (50 mg/kg) were completely protected. When HGN194 was given at 1 mg/kg, one out of two monkeys remained aviremic, whereas the other had delayed, lower peak viremia. Interestingly, all protected monkeys given high-dose HGN194 developed Gag-specific proliferative responses of both CD4+ and CD8+ T cells. To test whether generation of the latter involved cryptic infection, we ablated CD8+ cells after HGN194 clearance. No viremia was detected in any protected monkeys, thus ruling out virus reservoirs. Thus, induction of CD8 T-cell immunity may have resulted from transient “Hit and Run” infection or cross priming via Ag-Ab-mediated cross-presentation. Together, our data identified the HGN194 epitope as protective and provide proof-of-concept that this anti-V3 loop mAb can prevent infection with sterilizing immunity after challenge with virus of a different clade, implying that V3 is a potential vaccine target.

## Introduction

More than two decades after the discovery of the human immunodeficiency virus (HIV), developing an anti-HIV vaccine remains a crucial challenge. HIV clade C (HIV-C) comprises approximately 56% of all cases of HIV/AIDS worldwide (www.unaids.org) and predominates in sub-Saharan Africa, India and China, where it is found as B'/C recombinant virus with an HIV-C envelope. Thus, developing a safe and effective vaccine against this most prevalent HIV-1 subtype remains an important task.

Classical prophylactic vaccine approaches that successfully control various viral diseases are typically based upon neutralizing antibodies (nAbs). The first attempt to develop an anti-HIV-1 vaccine involved monomeric gp120. However, broad nAbs were not induced, and sera from vaccinated individuals failed to neutralize most primary HIV-1 isolates [1]. Two phase III trials using HIV-1 gp120 immunogens showed no protection [2], [3]. Interest in developing nAb-based AIDS vaccines was renewed by successful passive immunization studies in macaque models using broadly reactive human neutralizing monoclonal antibodies (bnmAbs) against challenge with chimeric simian-human immunodeficiency viruses (SHIVs) encoding HIV-1 envelope genes in an SIV backbone [4]–[12]. These studies provided proof-of-concept that full protection against primate immunodeficiency virus challenge could be achieved with bnmAbs targeting conserved, functionally important HIV-1 Env epitopes.

Initially, antibodies isolated from HIV-1 clade B-infected individuals targeting the third variable loop (V3) of HIV-1 gp120 were thought to be narrowly focused and strain-specific, due to high V3 sequence variability. However, V3 contains conserved structural elements involved in crucial interactions with coreceptors [13]; indeed, the V3 loop crown is thought to be organized into a folded domain that forms the basis for the cross-reactivity of some V3-specific mAbs, including 447-52D, 2219, 3014 and HGN194 [14]. Moreover, two potent bnmAbs, PG9 and PG16, have been discovered recently; both target highly conformational, discontinuous epitopes involving the V2 and V3 loops [15]. These data highlight the importance of V3 as target for broadly reactive nAbs.

The human anti-V3 mAb, HGN194 [16], isolated from memory B cells of a long-term non-progressor infected with a HIV-1 clade AG circulating recombinant form (CRF), targets an epitope in the V3-loop crown and neutralizes a range of relatively neutralization-sensitive and resistant viruses from clades A, B, C as well as recombinant AG and BC [16]. In this study, the IgG1 mAb HGN194 neutralized all tier 1 viruses, which are highly neutralization sensitive, and 11% of the tier 2 viruses tested. Tier 2 strains are more difficult to neutralize and reflect the majority of primary HIV-1 isolates.

Here, we evaluated the potential of HGN194 to protect rhesus monkeys (RM) against mucosal challenge with a heterologous SHIV encoding a CCR5-tropic (R5) HIV-C envelope. We found that at a high nmAb dose, all animals were completely protected, indicating for the first time potent cross-clade protection by a human anti-HIV-1 mAb in vivo. Interestingly, all SHIV-challenged RM treated with high-dose HGN194 developed Gag-specific T-cell immunity, although we found no evidence of virus reservoirs after HGN194 had cleared and the CD8+ cells were ablated with a cytotoxic mAb in protected RM. Thus, passive immunization with HGN194 is to our knowledge the first study that provided evidence of complete cross-clade protection.

## Results and Discussion

**Figure 1:**
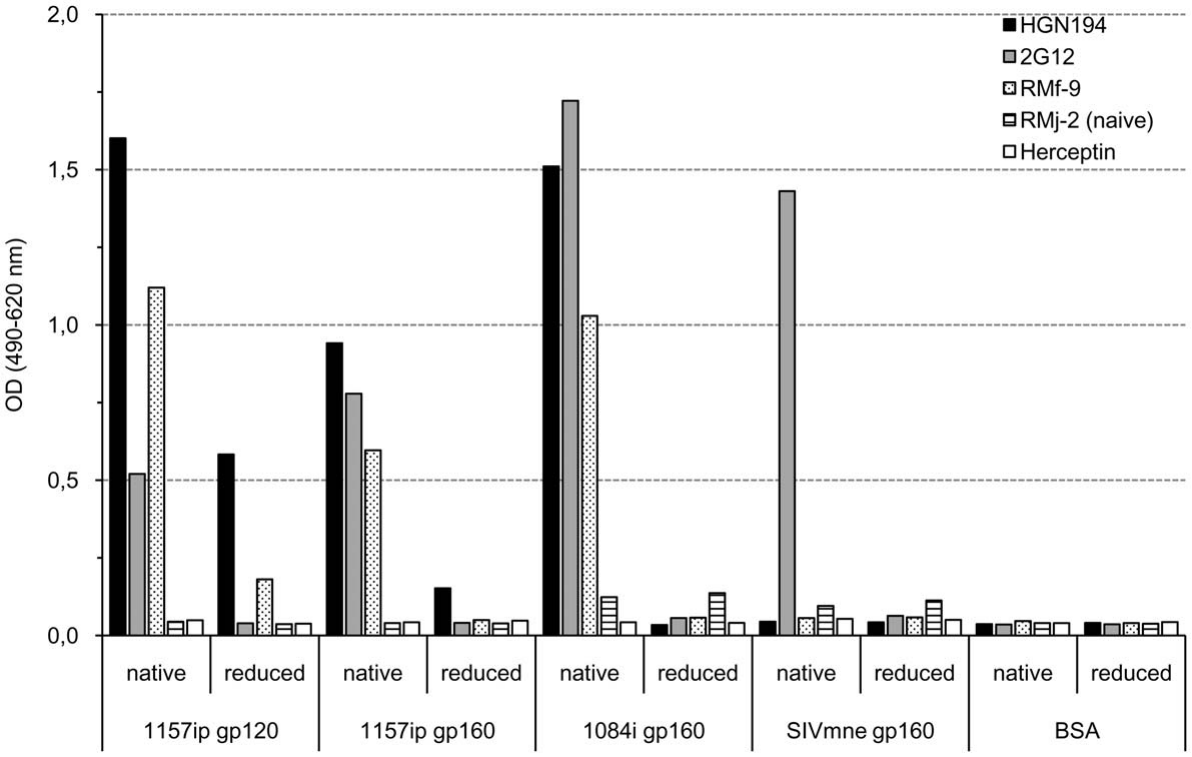
HGN194 targets a unique conformational V3-loop epitope. Plasma from monkey RMf-9 (white bars with black dots), chronically infected with SHIV-1157ip, was used as positive control as well as 2G12 (grey bars), a mAb targeting a conformational mannose-dependent epitope on gp120. Herceptin (white bars) and plasma from naïve monkey RMj-2 (striped bars) were used as negative controls. Results of conformational ELISA are shown. Additional control studies have been published earlier [24].

Given the diversity of V3 amino-acid sequences of viruses neutralized by HGN194 [16], we hypothesized that HGN194 recognizes a conformational epitope in the V3 crown. We thus evaluated HGN194 binding to gp120 and gp160 under native and reduced conditions (Figure 1). Compared to native Env, HGN194 binding was clearly decreased when either gp120 or gp160 were denatured, indicating conformation dependence of the HGN194 epitope.

We then evaluated the ability of HGN194 to neutralize a panel of R5-tropic SHIVs with different neutralization sensitivity in vitro (Table 1). SHIV-1157ipd3N4 is a “late”, tier 2 virus derived from a monkey after it developed AIDS [17], whereas SHIV-1157ipEL-p [18] is a chimera of the “early” SHIV-1157ip *env* (derived from a recently infected Zambian infant's primary R5 HIV-C) and the “late”, engineered backbone of SHIV-1157ipd3N4. The final SHIV-1157ipEL-p chimera was derived by rapid passage through four rhesus macaques for adaptation and has a tier 1 neutralization phenotype [18]. SHIV-1157ipd3N4 and SHIV-1157ipEL-p are isogenic forms of the same virus, differing only in the nature of their R5 HIV-C Envs. SHIV_SF162P4_ was derived from an R5 clade B HIV-1 and has a tier 1 neutralization-sensitive profile [19].

**Table 1:** Neutralization of clade B and C SHIV strains in TZM-bl and human PBMCs.

			TZM-bl-based assay				PBMC-based assay			
Virus	Tier a	HGN194 epitope	HGN194 (µg/ml)		4X (µg/ml) b		HGN194 (µg/ml)		4X (µg/ml) b	
		V3 sequence	IC_50_	IC_90_	IC_50_	IC_90_	IC_50_	IC_90_	IC_50_	IC_90_
HIV CRF02_AG c	2	RRSVRIGPGQTF	N.A.	N.A.	N.A.	N.A.	N.A.	N.A.	N.A.	N.A.
SHIV_SF162P4_	1	RKSITIGPGRAF	0.2	1.6	7.8	31.5	<0.04	1.3	0.8	15.8
SHIV-1157ipEL-p	1	RKSIRIGPGQAF	0.6	10.8	36	>40	0.01	0.09	0.9	>40
SHIV-1157ipd3N4	2	RKSISIGPGQAI	>40	>40	>40	>40	>40	>40	8.2	>40

a Neutralization tier assignment.

b 4X, quadruple combination of IgG1b12, 2G12, 2F5, and 4E10 at 1∶1∶1∶1 ratio.

c Parental virus that was not available for testing. The epitope is given for comparison.

HGN194 potently neutralized the tier-1 SHIV-1157ipEL-p and SHIV_SF162P4_ in vitro at low 50 and 90% inhibitory concentrations (IC_50_ and IC_90_, respectively). However, it failed to neutralize the tier 2 SHIV-1157ipd3N4 (Table 1).

**Figure 2:**
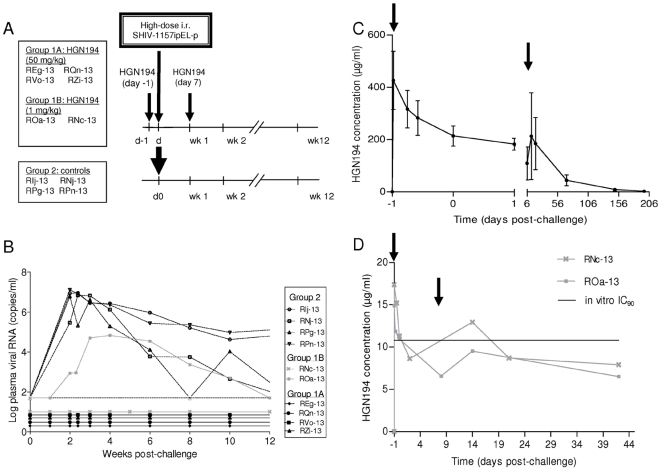
Passive immunization with HGN194 against heterologous clade C SHIV challenge in infant rhesus macaques. (A) Experimental design. Group 1A infant RM (n = 4) were infused twice with 50 mg/kg of HGN194 on days −1 and 7. Group 1B (n = 2) received twice 1 mg/kg of HGN194. Four RM served as untreated controls. On day 0, all 10 animals were challenged intrarectally with 18 AID_50_ of SHIV-1157ipEL-p. (B) Plasma viral RNA loads after high-dose rectal challenge with SHIV-1157ipEL-p using a quantitative RT assay (detection limit: 50 copies/ml). (C) Average plasma mAb concentrations during the course of the study for Group 1A. (D) Plasma HGN194 concentration for Group 1B. Arrows in C and D indicate mAb treatments. Experiments in C and D were repeated twice or trice.

Next, we sought to test the efficacy of HGN194 in vivo and enrolled two groups of infant RM. Group 1A (n = 4) was treated with HGN194 at 50 mg/kg. Group 2 control monkeys (n = 4) were left untreated. MAb treatments were given 24 h before and 7 days after virus challenge (Figure 2A). On day zero, all monkeys were challenged intrarectally with a high dose of SHIV-1157ipEL-p (18 50% animal infectious doses (AID_50_)). All controls (Group 2) became highly viremic with a mean peak viral RNA load of 8.6×10^6^±3.1×10^6^ copies/ml at week 2 (Figure 2B); in contrast, all infants of Group 1A remained aviremic throughout the course of the study. Western blots were positive for SIV Gag in all controls but negative in all Group 1A monkeys (data not shown). Similarly, lymph node biopsies (week 10) were negative for all four Group 1A infants but highly positive for Group 2 monkeys when tested by RT-PCR specific for SIV *gag* (Figure S1B). We conclude that HGN194, isolated from an HIV-positive individual harboring an AG CFR, was able to confer complete cross-clade protection against a clade C SHIV.

To estimate the minimal effective dose required for full protection, we performed a pilot study with Group 1B (n = 2) that received a 50-fold lower HGN194 dose. One animal (RNc-13) remained aviremic, while the second animal, ROa-13, became infected but showed a>100-fold lower peak viremia which was delayed by 2 weeks compared to controls (Figure 2B). Animal ROa-13 developed anti-SIV Gag antibodies, while RNc-13 did not (data not shown).

The animals were followed prospectively by clinical examination and analysis of T-cell subsets. HGN194 was well tolerated without obvious side effects. The absolute CD4 T-cell counts of the virus-exposed, uninfected animals of Group 1A and monkey RNc-13 showed normal, age-related declines also seen in human infants. At week 23, SHIV-1157ipEL-p-viremic controls had lower CD4 T-cell counts compared to mAb-treated, uninfected animals (Figure S1A).

Next, we sought to link nAb titers achieved in vivo with the degree of protection. First, we assessed HGN194 plasma concentrations by ELISA in mAb-treated monkeys. In Group 1A, the average HGN194 concentration was 213.7 µg/ml on the day of challenge (Figure 2C). HGN194 followed a biphasic decay with a mean half-life of 24.6±5.3 h in the first phase and a mean half-life of 31.4±9.2 days in the second phase. In Group 1B, the average HGN194 concentration on the day of challenge was 11.1 µg/ml (Figure 2D), which approximated the *in vitro* IC_90_ (10.8 µg/ml) observed in the TZM-bl neutralization assay with purified HGN194 *in vitro* (Table 1). Second, we measured the neutralizing capacity of monkey plasmas by TZM-bl assay against the challenge virus (SHIV-1157ipEL-p). When IC_50_ and IC_90_ values from individual monkeys were plotted against the plasma HGN194 concentrations, a correlation with IC_50_ (p = 0.0002) and IC_90_ (p = 0.0012) was observed in Group 1A (data not shown). From these data, we extrapolated an average *in vivo* IC_50_ of 0.2 µg/ml and an average *in vivo* IC_90_ of 2.15 µg/ml – values that were in the same order of magnitude as the initial in *vitro* IC_50_ (0.6 µg/ml) and IC_90_ (10.8 µg/ml) obtained by TZM-bl assay against the challenge virus (Table 1). These data suggest a direct relationship between the in vitro inhibitory concentrations and protection from challenge in an in vivo model. This result is in accordance with the recent finding that mAb 2G12 serum neutralizing titers of the order of 1∶1 (IC_90_) can protect animals with sterilizing immunity and contrasts strongly with the high titers needed for protection by other nAbs, including the bnmAb b12 [11]. This may be linked to the lack of autoreactivity of both HGN194 and 2G12 [20] (Figure S2) and/or differences in the mechanisms of neutralization.

**Figure 3:**
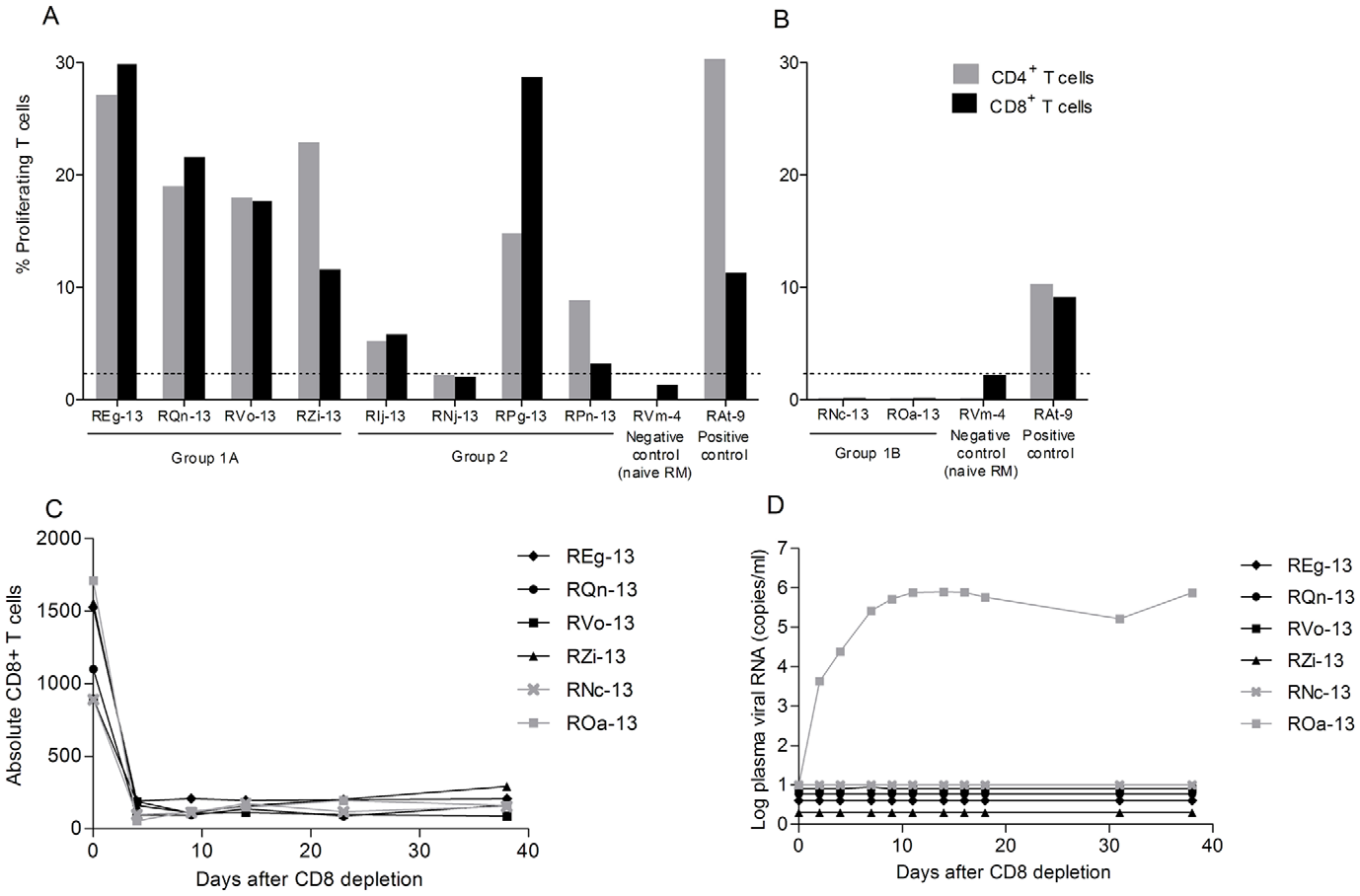
Induction of specific antiviral T-cell responses after passive immunization. (A) and (B) Proliferation of T cells. PBMC were stimulated with SIVmac251 Gag protein and proliferation of CD4^+^ and CD8^+^ cells was measured using the CFSE dilution method. (A) Animals given 50 mg/kg of HGN194 (Group 1A) and control group RM (Group 2). PBMC collected 17 weeks post-challenge were tested. Horizontal dashed line, cut-off value based on response shown by a naïve animal. (B) Animals given 1 mg/kg of HGN194 (Group 1B). PBMC collected at weeks 12 (RNc-13) and 17 (ROa-13) post-challenge were tested. (C) Absolute numbers of CD8+ T cells for Groups 1A (black) and 1B (grey) after treatment with a cytotoxic anti-CD8 mAb (Methods). (D) Plasma viral RNA load after CD8+ cell depletion.

We then sought to test whether the macaques protected by passive immunization had developed antiviral cellular immune responses, given the likely development of antigen-antibody complexes. We performed interferon-γ ELISPOT assays after stimulation of PBMC with SIV Gag, Nef and HIV-1 Tat peptides, as well as T-cell proliferative assays by CFSE dilution after stimulation with SIV Gag and HIV-1 Env and Tat proteins. All mAb-treated animals were ELISPOT negative, in contrast to Group 2 controls (3 out of 4 RM had total spot-forming units ranging from 130 to 480/million cells; data not shown). However, all Group 1A monkeys showed proliferation of CD4^+^ as well as CD8^+^ T cells after stimulation with SIV Gag (Figure 3A) but not with HIV-1 Env and Tat proteins (data not shown), suggesting that specific memory T cells developed only against the most abundant virion protein, SIV Gag. Of note, all RM of the virus-only control Group 2 also had proliferative responses in the CD4+ and CD8+ T-cell populations, although the levels differed among the individual monkeys (Figure 3A). Next, we tested Group 1B, which had received a 50-fold lower HGN194 dose, for proliferative T-cell responses to viral proteins; no T-cell responses to any viral proteins were detectable (Figure 3B). Thus, only RM exposed to virus in the context of a high but not a low nmAb dose developed Gag-specific proliferative responses of both CD4+ and CD8+ T cells.

The following mechanisms may have induced virus-specific CD8+ T cells in the persistently aviremic monkeys: a) cryptic infection with persistent reservoir cells capable of releasing infectious after loss of immune control; b) transient, cryptic “Hit and Run” infection without reservoir cells but providing sufficient antigen exposure to generate specific cellular immunity; or c) cross-presentation of antigen-antibody complexes in the absence of target cell infection [21].

To test for the possibility of virus-producing reservoir cells, we depleted CD8+ T and NK cells with a rhesus anti-CD8 mAb in all Group 1A and 1B monkeys after HGN194 plasma concentrations had declined below the IC_50_. A rapid drop of CD8+ T cells was accompanied by a rise in viremia for ROa-13, the Group 1B monkey that initially had delayed lower viremia (Figure 3C and 3D). In contrast, all protected monkeys from Groups 1A and 1B remained aviremic after CD8+ cell depletion, indicating that no viral reservoir capable of releasing virus existed. Currently, we cannot differentiate between a transient cryptic “Hit and Run” infection that resolved without leaving behind any infected reservoir cells or cross-presentation of antigen-antibody complexes, although the latter is an attractive hypothesis, especially in light of the fact that Group 1B monkeys had not developed any T-cell proliferative responses. This might be ascribed to lower levels of antigen-antibody complexes formed after treatment with the 50-fold lower HGN194 dose compared to Group 1A RM.

In general, in vitro neutralization assays reflect inhibition of viral entry. However, nmAb effector functions, such as antibody-dependent cellular cytotoxicity (ADCC) or complement activation, may also contribute to the overall antiviral activity in *vivo*. Effector-deficient mAb mutants have been used to demonstrate the importance of these mechanisms in macaques [12]. ADCC activity of HGN194, an IgG1 nmAb, may have contributed to the complete protection from systemic infection in our study – perhaps via eradication of small numbers of infected target cells, thus allowing a “Hit and Run” transient infection that primed T-cell immunity. Passive immunization studies with effector function- deficient HGN194 mutants would assess the relative contribution of standard virus neutralization versus mAb effector functions to the protective action of HGN194.

In summary, we have shown that HGN194, an nmAb targeting a unique conformational epitope, can confer complete protection against a heterologous, high-dose mucosal clade C SHIV challenge in macaques. These results identify the HGN194 target as a protective epitope *in vivo* and a potential vaccine target for cross-clade immunization.

## Materials and Methods

### Cell lines, viruses and antibodies

TZM-bl cells were purchased from the NIH AIDS Research and Reference Reagent Program (ARRRP). HGN194 was prepared as described [16]. The SHIV-1157ipEL-p stock (grown in RM PBMC) had a p27 concentration of 50 ng/ml and 1.5x10^5^ 50% tissue culture infectious doses (TCID_50_)/ml as measured in TZM-bl cells.

### In vitro neutralization assays

Heat-inactivated, serially diluted plasmas/antibodies were incubated with virus for 1 h at 37°C and then added to cells. The TZM-bl assay was performed as described [22]. PBMC assays were performed as published [23], except that polymyxin B (15 µg/ml) was added to block potentially present endotoxin. Throughout the assay, the mAbs were not washed away but diluted 1∶1 with fresh medium daily, starting on day 3. Supernatant aliquots were harvested every other day and assayed for p27 levels in wells containing only virus+cells. Neutralization was measured on the culture day showing a linear phase of increase in control wells.

### Conformational ELISA

ELISA was performed as described [24] with modifications. Plates were coated with 40 ng/well of native or reduced (20 mM TCEP/0.4% SDS; boiled for 5 min) proteins and the next day washed, blocked and incubated overnight at 4°C with 100 µl/well of either HGN194, Herceptin, 2G12 (5 µg/ml in PBSCT) or RM serum (1∶400; 100 µl/well diluted in PBSCT). After washing, plates were incubated with HRP-conjugated antibodies, washed 10 times, developed and read at 490/620 nm.

### Animal care and passive immunization

This study was carried out in strict accordance with the recommendations in the Guide for the Care and Use of Laboratory Animals of the U.S. Public Health Services/National Institutes of Health, as well as according to the recommendations in the Weatherall report on “The Use of Non-human Primates in Research” (http://www.acmedsci.ac.uk/images/project/nhpdownl.pdf). The protocol was approved by the Committee on the Ethics of Animal Experiments of Emory University (IACUC ID: 027-2009Y; Emory University Animal Welfare Assurance Number A3180-01). The rhesus monkeys were housed at the Yerkes National Primate Research Center (YNPRC, Emory University, Atlanta, GA). YNPRC facilities are fully accredited by the Association for Assessment and Accreditation of Laboratory Animal Care International. Animal experiments were approved by the Institutional Animal Care and Use Committees at Emory and the Dana-Farber Cancer Institute via a Collaborating Institution Animal Use Agreement. Because the experiments described here involved a virus that may cause an incurable disease, such as AIDS, discomfort, stress and pain may occur. Animals were closely monitored and observed for development of disease at least twice daily. If the animals are determined to be under stress or in discomfort, appropriate anesthetics and/or analgesics are administered as directed by the clinical veterinary staff. Euthanasia is also an option should treatment not alleviate stress. In the current study, no untoward clinical problems were noted, and none of the virus-infected monkeys progressed to AIDS.

Four RM received two i.v. doses of HGN194 (50 mg/kg); two monkeys received 1 mg/kg. Four untreated animals served as controls. All monkeys were challenged intrarectally with 18 50% animal infectious doses (AID_50_) of SHIV-1157ipEL-p.

### Assessment of plasma viral RNA levels

Plasma viral RNA levels were measured as described [18], [25].

### Lymphocyte proliferation assay

The assay was performed as described [26]. In brief, PBMC were stained with CFSE and incubated with/without antigen (SIV Gag, HIV-1 Tat or SHIV-1157ip gp160; 2 µg/ml) for 5 days at 37°C. Cells were stained with anti-CD3-Alexa Fluor 700 (clone SP34-2), anti-CD4-PerCP (clone L200) and anti-CD8-PE (clone RPA-T8) antibodies. The percentage of proliferating CD3^+^CD4^+^ and CD3^+^CD8^+^ cells was determined using FACSDiva (BD Biosciences) software.

### Anti-CD8 antibody administration

M-T807R1 (Nonhuman Primate Reagent Resource, Beth Israel Deaconess Medical Center, Boston, MA), a primatized anti-CD8 mAb, was administered subcutaneously (50 mg/kg) on day 0. MAb administration was initiated 7 months after SHIV-1157ipEL-p challenge for Group 1A, while monkeys ROa-13 and RNc-13 (Group 1B) were given mAb at 5.5 and 4 months post-challenge, respectively.

### HGN194 plasma concentration

HGN194 plasma concentrations were determined by ELISA. Nunc 96-well plates were coated with gp120 of SHIV-1157ip (2 µg/ml) and incubated overnight at 4°C. After blocking and washing, serially diluted plasmas were added. HGN194 was included as standard ranging from 1.5 to 0.046 ng/ml. After washing, plates were incubated 1 h with an anti-human IgG HRP antibody (Zymed, San Francisco, CA). After washing, HRP substrate (Rockland Immunochemicals, Gilbertsville, PA) was added and after 30 min, the reaction was stopped with 1 N sulfuric acid and the OD was measured at 450 nm with a microplate reader (Berthold, Oak Ridge, TN). To determine the half-life of HGN194, natural logs of HGN194 plasma levels were plotted as a function of time from the end of infusion. Slopes of the linear graphs were determined by least-squares analysis. Half-lives were calculated as t_1/2_ = −(ln 2)/m.

### Statistical analysis

Statistical analyses were performed using Graph Pad Prism for Windows, version 5 (Graph Pad Software Inc., San Diego, CA).

## Supporting Information

Figure S1 **Absolute CD4+ T cells and lymph node biopsies.** (A) Absolute CD4 T cell counts. From week 23 onward, the SHIV-1157ipEL-p-infected control monkeys showed decreased CD4 T-cell counts compared to uninfected animals. (B) Lymph node biopsies (week 10) were negative for all four Group 1A infants but highly positive for Group 2 monkeys when tested by RT-PCR specific for SIV *gag*. Black bars, RT-PCR performed with 1 µg of RNA per reaction; stippled grey bars, 0.25 µg of RNA per RT-PCR test.
(TIF)

Figure S2 **HGN194 lacks of autoreactivity.** MAbs were tested for their autoreactivity by indirect immunofluorescence using the Biochip mosaic diagnostic assay (Euroimmun, Germany). All mAbs were tested at 10 µg/ml using Hep-20-10 cells for antinuclear antibodies, primate liver for antibodies directed against liver-specific proteins and liver-cell membranes, rat kidney for antibodies targeting kidney microsomes or mitochondria, and rat stomach for antibodies binding to smooth muscle. MAb 2F5 was included because Haynes et al. [16] demonstrated its autoreactivity. In the same study, 2G12 had no evidence of autoreactivity. In contrast to 2F5, 2G12 was found to have a long half-life (Armbruster C, et al. A phase I trial with two human monoclonal antibodies (hMAb 2F5, 2G12) against HIV-1. AIDS 2002; 16: 227–233). In Figure S2, mAbs 2G12 and HGN194 did not show autoreactivity, while the mAb 2F5 did. The irregular staining in monkey liver sections is considered non-specific.
(TIF)
