# Supplementary figures and images for: An Anti-HIV-1 V3 Loop Antibody Fully Protects Cross-Clade and Elicits T-Cell Immunity in Macaques Mucosally Challenged with an R5 Clade C SHIV
Source: PLoS One. 2011 Mar 31;6(3):e18207. doi: 10.1371/journal.pone.0018207 (PMC3069056; doi:10.1371/journal.pone.0018207)

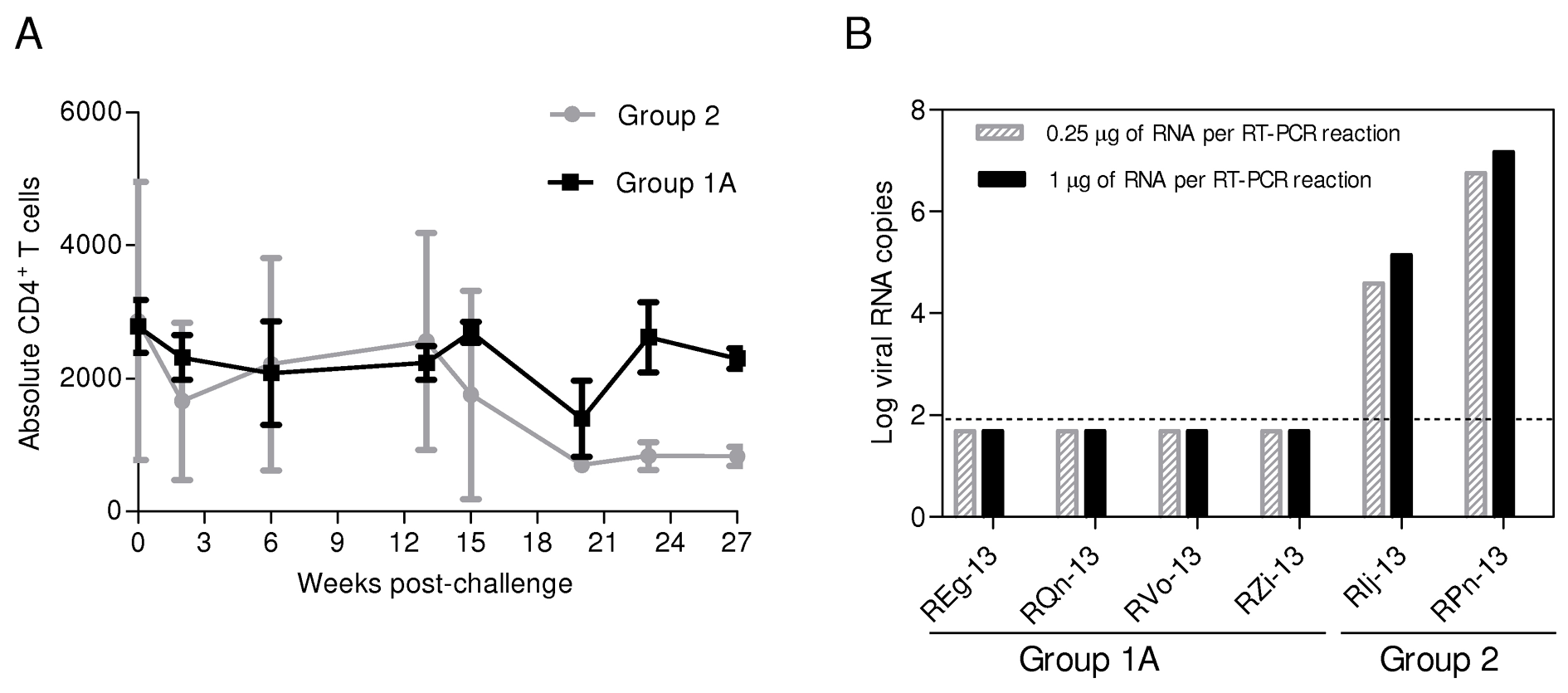

Supplement: Figure S1 — Absolute CD4+ T cells and lymph node biopsies. (A) Absolute CD4 T cell counts. From week 23 onward, the SHIV-1157ipEL-p-infected control monkeys showed decreased CD4 T-cell counts compared to uninfected animals. (B) Lymph node biopsies (week 10) were negative for all four Group 1A infants but highly positive for Group 2 monkeys when tested by RT-PCR specific for SIV gag. Black bars, RT-PCR performed with 1 µg of RNA per reaction; stippled grey bars, 0.25 µg of RNA per RT-PCR test. (TIF) [file pone.0018207.s001.tif]

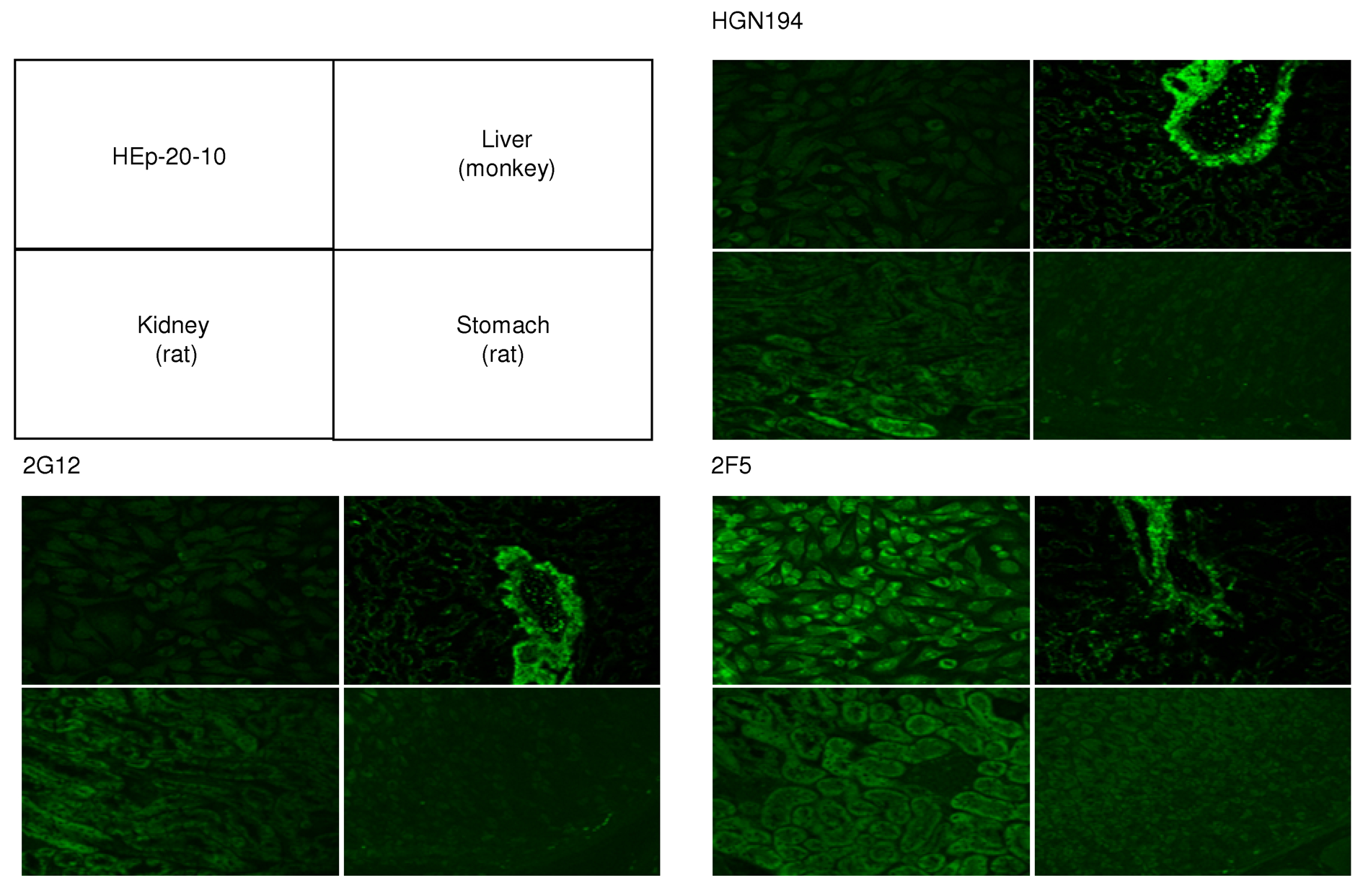

Supplement: Figure S2 — HGN194 lacks of autoreactivity. MAbs were tested for their autoreactivity by indirect immunofluorescence using the Biochip mosaic diagnostic assay (Euroimmun, Germany). All mAbs were tested at 10 µg/ml using Hep-20-10 cells for antinuclear antibodies, primate liver for antibodies directed against liver-specific proteins and liver-cell membranes, rat kidney for antibodies targeting kidney microsomes or mitochondria, and rat stomach for antibodies binding to smooth muscle. MAb 2F5 was included because Haynes et al. [16] demonstrated its autoreactivity. In the same study, 2G12 had no evidence of autoreactivity. In contrast to 2F5, 2G12 was found to have a long half-life (Armbruster C, et al. A phase I trial with two human monoclonal antibodies (hMAb 2F5, 2G12) against HIV-1. AIDS 2002; 16: 227–233). In Figure S2, mAbs 2G12 and HGN194 did not show autoreactivity, while the mAb 2F5 did. The irregular staining in monkey liver sections is considered non-specific. (TIF) [file pone.0018207.s002.tif]
